# Differential Effects of Retinoic Acid Concentrations in Regulating Blood–Brain Barrier Properties

**DOI:** 10.1523/ENEURO.0378-16.2017

**Published:** 2017-05-26

**Authors:** Stephanie Bonney, Julie A. Siegenthaler

**Affiliations:** Department of Pediatrics, Section of Developmental Biology School of Medicine Aurora, University of Colorado, CO 80045

**Keywords:** blood brain barrier, brain vascular development, LXR, RXR, endothelial cell

## Abstract

The blood-brain barrier (BBB) is a multifaceted property of the brain vasculature that protects the brain and maintains homeostasis by tightly regulating the flux of ions, molecules, and cells across the vasculature. Blood vessels in the brain are formed by endothelial cells that acquire barrier properties, such as tight and adherens junctions, soon after the brain vasculature is formed. Endothelial WNT signaling is crucial to induce these BBB properties by regulating their expression and stabilization. Recent studies have implicated retinoic acid (RA) signaling in BBB development and shown that pharmacological concentrations of RA (≥5 µm) can induce BBB properties in cultured brain endothelial cells. However, a recent study demonstrated that RA inhibits endothelial WNT signaling during brain development, suggesting that RA does not promote BBB properties. We therefore investigated whether RA plays a physiological role in BBB development. We found that BBB function and junctional protein expression was unaffected in mouse mutants that have a reduced capacity to synthesize RA (*Rdh10* mutants). Furthermore, embryos exposed to a RA-enriched diet did not enhance BBB protein expression. Together, our data indicate that RA is not capable of inducing, nor is it required for, BBB protein expression *in vivo*. Like other studies, we found that pharmacological concentrations of RA induce BBB genes in cultured murine brain endothelial cells, and this may involve activation of the LXR/RXR signaling pathway. Our data do not support a role for RA in BBB development, but confirm reports that pharmacological RA is a robust tool to induce BBB properties in culture.

## Significance Statement

Uncovering signals that promote BBB properties in CNS blood vessels is crucial to understand how the brain vascular network supports brain function. In contrast to previous studies, we provide substantial evidence that RA signaling is not required for prenatal BBB development. However, we show that RA at pharmacological concentrations (≥5 μm) is a useful tool to promote BBB properties in brain ECs and that this effect may be due to LXR/RXR signaling. We have also revealed a potential independent function of LXRs in regulating the expression of BBB genes. These studies could provide insight into mechanisms that underlie the BBB breakdown that occurs in many CNS diseases and improve *in vitro* BBB models to study drug delivery and BBB biology.

## Introduction

Endothelial cells (ECs) that form blood vessels are specialized in the brain to tightly regulate the transportation of molecules, ions, and cells into and out of the CNS. Various properties are acquired by the brain ECs to form the blood–brain barrier (BBB), the main being an enrichment of tight junctions (TJs) and adherens junctions (AJs). TJs and AJs work together to seal and strengthen the intercellular connections between brain ECs to form a continuous endothelium. The integrity of these junctional complexes is crucial to provide a permissive environment for neuronal function by regulating ionic homeostasis and allowing for effective neuronal action potentials. Furthermore, TJs limit the infiltration of neurotoxic compounds, pathogens, and immune cells, thus preventing CNS injury and disease. Disruption of BBB junctions can affect oxygen and nutrient supply, disrupt ionic homeostasis, and accelerate immune invasion into the CNS. The BBB is also a major obstacle for drug delivery and proper treatment of various neurologic disorders (Hawkins and Davis 2005; Obermeier et al. 2013; Bauer et al. 2014; Engelhardt and Liebner 2014). Therefore, many studies have focused on developing *in vitro* BBB models to identify drugs that can effectively pass the BBB. However, the molecular cues that promote BBB properties are not fully understood.

CNS ECs obtain BBB properties soon after the blood vessels establish an immature vascular network. In mice, these properties are acquired at approximately embryonic day 13 (E13; Obermeier et al. 2013; Siegenthaler et al. 2013). Although both VEGF and WNT signaling promote angiogenesis of the CNS vasculature, WNT signaling is required for BBB development and maintenance. The WNT transcriptional effector, β-catenin, regulates the expression of TJ proteins Claudin-3 and Claudin-5 in brain ECs. Additionally, β-catenin is required for stabilization of AJs by interacting with VE-cadherin (Liebner et al. 2008; Zhou and Nathans 2014; Zhou et al. 2014).

Recent studies have emerged suggesting a possible role for retinoic acid (RA) signaling in the development of the BBB (Mizee et al. 2013). RA is synthesized from Vitamin A through a series of enzymatic steps that ultimately yield all-trans RA (atRA) and 9-cis RA (9cRA). AtRA binds and activates nuclear receptors called retinoic acid receptors (RARs) with high affinity, whereas 9cRA has high affinity for the retinoid X receptors (RXRs; Tanoury et al. 2013). Mizee et al. (2013) identified astrocytes as a potential source of RA that can induce BBB properties in human brain ECs *in vitro.* They found that embryos treated with an RAR inhibitor during development displayed increased BBB permeability and reduced vascular expression of the TJ protein ZO-1. Furthermore, these and other studies showed that pharmacological concentrations of atRA (≥5 μm) can induce TJ and AJ protein expression in both human brain ECs (Mizee et al. 2013) and induced pluripotent stem cell (iPSC)-derived human brain ECs (Lippmann et al. 2014; Katt et al. 2016). Mizee et al. (2013) based their conclusion that RA plays a physiologic role in BBB development in part on the observation that pharmacological concentrations of RA induced BBB gene expression *in vitro*. The pharmacological concentrations of atRA used in these studies, while eliciting an effect on BBB properties, are well above what is considered the physiologic range of RA, which is ∼2–600 nm (Napoli et al. 1991). Therefore, it is unclear whether RA plays a physiologic role in BBB development. Furthermore, a recent study showed that RA inhibits endothelial WNT signaling during brain vascular development (Bonney et al. 2016), suggesting that RA could limit BBB properties. To understand these discrepancies, we used *Rdh10* mutants, which have a reduced capacity to synthesize RA, to investigate a physiologic role for RA in BBB development. We observed that BBB function and junctional protein expression are retained in *Rdh10* mutants. Moreover, embryos from pregnant females exposed to an RA-enriched diet did not display enhanced BBB features. Instead, we found a reduction in BBB protein expression; however, BBB leakage was not observed, suggesting that RA exposure does not overtly affect barrier function. Collectively, these data do not support a physiologic role for RA in BBB development. Conversely, we found that pharmacological concentrations of RA induced BBB gene expression in murine brain endothelioma cells, and this effect may be working through complex signaling events that involve LXR/RXR activity.

## Methods

### Animals and RA-enriched diets

Mice used for experiments were housed in specific pathogen–free facilities approved by the American Association for Laboratory Animal Care and were handled in accordance with protocols approved by the animal care committee regulations at the University of Colorado, Denver. The *Rdh10* ENU point mutation mutant allele has been described previously (Ashique et al. 2012), and these animals were obtained from Andy Peterson at Genentech. RA-enriched diet (final concentration 0.175 mg/g food) consisted of atRA (Sigma-Aldrich) dissolved in corn oil and mixed with Bioserv Nutra-Gel Diet, Grain-Based Formula, Cherry Flavor. atRA diet was prepared fresh daily and provided *ad libitum* to pregnant wild-type females beginning in the afternoon of E10 through the day of collection on E16.

### Immunohistochemistry

*Rdh10* mutants (E13.5–E14.5; *n* = 3), RA-exposed embryos (E16.5; *n* = 5), and their respective controls (E13.5–E14.5: *n* = 3; E16: *n* = 5) were collected, and whole heads or brains were fixed overnight in 4% paraformaldehyde. All tissues were cryoprotected with 20% sucrose in PBS and subsequently frozen in OCT. Tissue was cryosectioned in 12-μm increments. Immunohistochemistry was performed on tissue sections using the following antibodies: rabbit anti–Claudin-3 1:200 (Invitrogen), rabbit anti–Claudin-5 1:200 (Abcam), rabbit anti-fibrinogen 1:500 (Abcam), rat-anti PLVAP 1:100 (AbD Serotec), rabbit anti–VE-cadherin 1:200 (Abcam), and mouse anti–ZO-1 1:100 (Thermo Fisher Scientific). After incubation with primary antibodies, sections were incubated with appropriate Alexa Fluor–conjugated secondary antibodies (Invitrogen), Alexa Fluor 633–conjugated isolectin-B4 (Invitrogen), and DAPI (Invitrogen). For VE-cadherin staining, tissue was immediately frozen in OCT, cryosectioned, and fixed with methanol for 10 min before immunostaining. For Claudin-5 staining, antigen retrieval was performed on unfixed tissue, and Claudin-5 expression was detected using a tyramide signal amplification kit according to product specifications (Thermo Fisher Scientific). Immunofluorescent (IF) images were captured using a Zeiss 780 LSM confocal microscope, 40× objective with 2× optical zoom to reveal junctional organization. Laser percentage and gain settings were always set on the control tissue. Only few instances occurred where settings required slight reductions (*Rdh10* mutants for Claudin-3 and VE-cadherin) or increases (atRA-exposed brains for Claudin-5 and VE-cadherin) to obtain a clear signal.

### Immunoblots

Meninges were removed and forebrains (E16.5) from control (*n* = 8) or RA-exposed (*n* = 10) embryos were collected, homogenized, and lysed in TEN buffer [50 mm Tris, pH 7.5, 150 mm NaCl, 0.1% NP-40, 5 mm EDTA, 1 mm PMSF, and protease inhibitors (Roche)]. Protein concentration was determined using a BCA kit (Pierce). Lysates were combined with 4× sample buffer (300 mm Tris, 5% SDS, 50% glycerol, 0.025% bromophenol blue, and 250 mm β-mercaptoethanol) and protein (experiment 1, 15 μg; experiment 2, 50 μg) was run on Protean Tris-HCI 4%–20% gradient gel (Bio-Rad) then transferred onto nitrocellulose membranes (Bio-Rad) using the Trans-Blot Turbo System (Bio-Rad). Immunoblots were blocked with 5% nonfat dehydrated milk (NFDM) in Tris-buffered saline (TBS) with 0.1% Tween (TBS-T) for 1.5 h then incubated overnight at 4°C in 2.5% NFDM in TBS-T containing primary antibodies for rabbit anti–Claudin-3 1:250 (Invitrogen), rabbit anti–Claudin-5 1:1000 (Abcam), and rabbit anti–VE-cadherin 1:1000 (Abcam). After primary incubation, blots were washed and incubated in the 2.5% NFDM containing the appropriate horseradish peroxidase–linked secondary antibody (1:5000; Santa Cruz Biotechnology) for 1 h at room temperature. Clarity ECL substrate (Bio-Rad) and the ChemiDoc MP system (Bio-Rad) were used to visualize immunolabeled protein bands. Blots were stripped with stripping buffer (Restore Plus; Thermo Fisher Scientific) and reprobed with mouse anti–β-actin (1:2000; Cell Signaling Technology) antibody as a loading control. Densitometry of bands was performed using ImageLab software (Bio-Rad); density values were corrected for loading variations within each blot using the intensity of β-actin expression.

### Cell culture and pharmacological treatments

The mouse brain endothelioma cell line (bEnd3.1) was obtained from ATCC (CRL-2299). All experiments were performed on cells from passages 3–8 from when they were received (usually shipped from ATCC at passages within the low 20s), and cells were grown in Dulbecco’s minimal essential media with 4.5 g/L glucose, 1.5 g/L sodium bicarbonate, 4 mm l-glutamine (Invitrogen), 10% fetal bovine serum (FBS; Invitrogen), and penicillin-streptomycin (100 U/mL; Invitrogen). Cells were plated on collagen-coated (10 μg/cm^2^; Sigma) 12-well plates (VWR) for quantitative PCR (qPCR) analysis or four-well glass chambered slides (Nunc Lab-Tek) for immunocytochemistry. Once cells reached confluence, they were serum-starved overnight and treated for 24 h for mRNA expression analysis or 48 h for immunocytochemistry analysis with vehicle (DMSO), atRA, or 9cRA (50 nm, 1 μm, or 5 μm; Sigma-Aldrich) in serum-free media. To modulate LXR/RXR signaling *in vitro*, bEnd.3 cultures were treated with vehicle (DMSO), 100 nm GSK-2033 (LXR antagonist; Tocris) with and without 5 μm atRA, or 1 μm T0901317 (LXR agonist; Tocris) in serum-free DMEM for 24 h and RNA purified for qPCR and transcriptional analysis. Each treatment condition included three wells of bEnd.3 cells per experiment. Each experiment was repeated three times (*n* = 3) on two separate cryobatches. Data presented in the main figures are representative of one experiment.

### qPCR and expressional analysis

Meninges were removed from the nonneocortical brain regions of E14 wild-type or *Rdh10* mutants (*n* = 5), and RNA was isolated using the RNeasy Mini Kit (Qiagen). Similarly, RNA was isolated and qPCR was performed from cultured bEnd.3 cells after 24 h of treatment with DMSO, atRA, 9cRA, GSK-2033, or T0901317. To synthesize cDNA, specifications were followed using the iScript cDNA Synthesis Kit (Bio-Rad) with 1 µg of RNA from each sample. To assess transcript levels of *Cldn3*, *Cldn5*, *Cdh5,* and *Tjp1* in the tissue samples and *Cldn5*, *Cdh5, Tjp1, Rarb, Gpihbp1, Fabp4, Abcg1*, and *Apoe* in the cell culture experiments, qRT-PCR was performed according to the SYBR Green (Bio-Rad) protocol using the Bio-Rad CFX96 Real Time PCR Detection System. For an internal control, *Actb* transcript levels were also assessed. To identify expressional differences in control and mutant genotypes or vehicle and treated samples, delta-δ CT analysis was applied. Primer sequences (5′ to 3′) are as follows: *Abcg1* forward: TCCATCGTCTGTACCATCCA, *Abcg1* reverse: TACTCCCCTGATGCCACTTC; *Actb* forward: CTAGGCACCAGGGTGTGAT, *Actb* reverse: TGCCAGATCTTCTCCATGTC; *Apoe* forward: AACAGACCCAGCAAATACGC, *Apoe* reverse: ATGGATGTTGTTGCAGGACA; *Cdh5* forward: CAACTTCACCCTCATAAACAACCAT, *Cdh5* reverse: ACTTGGCATGCTCCCGAT; *Cldn3* forward: CAGACCGTACCGTCACCACT, *Cldn3* reverse: ATTCGGCTTGGACAGTTCCT; *Cldn5* forward: GCTCTCAGAGTCCGTTGACC, *Cldn5* reverse: ATCTAGTGCCCCCAGGATCT; *Fabp4* forward: TGTGATGCCTTTGTGGGAAC, *Fabp4* reverse: CGCCCAGTTTGAAGGAAATC; *Gpihbp1* forward: CCAGCCCATCATCAAGACAG, *Gpihbp1* reverse: GATGAGCAGCCTTGACAACC; *Rarb* forward: CCAGGAAACCTTTCCCTCAC*, Rarb* reverse: GAGCAGGGTGATCTGGTCTG; and *Tjp1* forward: GCCCTAAACCTGTCCCTCAG, *Tjp1* reverse: GCAGAAGGCTTGCTCTCAAA.

### Immunocytochemistry

bEnd.3 cultures were plated on collagen-coated (10 μg/cm^2^; Sigma-Aldrich) 12-well glass chambered slides (Nunc Lab-Tek) and treated with atRA or 9cRA (*n* = 3; DMSO, 50 nm, 1 μm, or 5 μm) in serum-free media for 48 h after they reached ∼80% confluence and were serum-starved overnight. The cultures were then washed with PBS and fixed with methanol for 10 min and immunolabeled with rabbit anti–VE-cadherin or mouse anti–ZO-1 (as above). After incubation with primary antibodies, samples were incubated with appropriate Alexa Fluor–conjugated secondary antibodies (Invitrogen) and DAPI (Invitrogen). IF images were captured using a Zeiss 780 LSM confocal microscope using methods as described above. Fluorescent intensity was determined using ImageJ (NIH) and normalized to the number of cells per 20× image.

### Statistics

Student *t* tests (parametric) and outlier tests (Grubbs’ test) were used to compare the differences between mean values of control (e.g., WT or vehicle) and experimental samples (e.g., Rdh10 mutant, single RA concentration) and to identify significant outliers within data sets, respectively (GraphPad; qPCR analysis, and fluorescence intensity). Student *t* tests with D’Agostino and Pearson normality (parametric) test were used to compare the differences of BBB protein expression between control and atRA treatment conditions from both experiments. To compare multiple mean values of different treatment conditions (Fig. 5*B*) to one another, ANOVA (parametric) was used followed by Tukey’s test. *p* < 0.05 was considered statistically significant. SD is reported on all graphs.

## Results

### BBB function and protein expression are not affected in the nonneocortical vasculature of Rdh10 mutants

**Figure 1.**
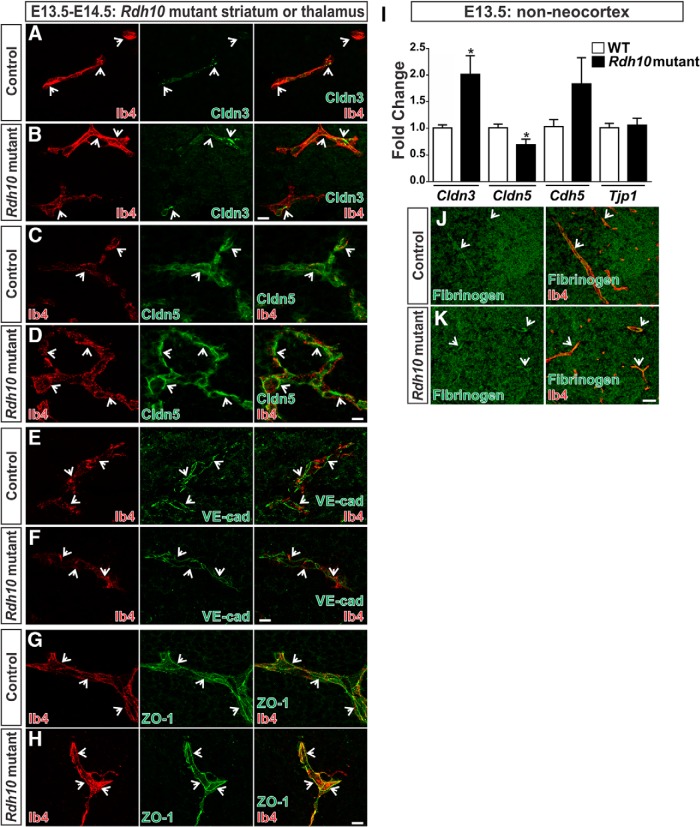
BBB function and protein expression are not affected in the nonneocortical vasculature of *Rdh10* mutants. ***A***, ***B***, E14.5 control (*Rdh10^+/+^* or *Rdh10* ^+/–^) and *Rdh10* mutant sections immunostained for Claudin-3 (green) and Ib4^+^ (red) to visualize blood vessels. Claudin-3 was observed in a punctate pattern in Ib4^+^ vessels in the striatum (arrows). ***C–H***, Arrows indicate Claudin-5 (***C*** and ***D***; green; thalamus), VE-cadherin (***E*** and ***F***; green; thalamus), or ZO-1 (***G*** and ***H***; green; striatum) colocalized with Ib4^+^ vessels (red) and junction formation in both E13.5 control and *Rdh10* mutant blood vessels. Scale bars are 10 µm. ***I***, Graph depicting transcript expression relative to control *Actb*, as determined by qPCR, for *Cldn3*, *Cldn5*, *Cdh5* (VE-cadherin), and *Tjp1* (ZO-1) in E14.5 control and *Rdh10* nonneocortices (*n* = 5; striatum, thalamus, midbrain, and hindbrain). Unpaired two-tailed *t* tests were performed; *, *p* < 0.05. ***J***, ***K***, IHC for fibrinogen (green) and Ib4^+^ vessels (red) on the striatum of E13.5 mice show retention of fibrinogen within the vasculature of control and *Rdh10* mutants (arrows). Scale bar is 50 µm.

*In vitro* BBB models have implicated RA signaling in promoting the expression of BBB proteins such as VE-cadherin, ZO-1, and Occludin (Mizee et al. 2013; Lippmann et al. 2014; Katt et al. 2016). However, it is unclear whether RA is required for BBB development. To test this, we assessed BBB protein expression in the brains of *Rdh10* mutant embryos. *Rdh10* mutants survive until E14.5 and have an ENU-induced point mutation in the RA-biosynthetic enzyme *Rdh10*, resulting in a diminished capacity to synthesize RA (Ashique et al. 2012), thus allowing us to assess whether RA is required for BBB development. Recent work identified defects in blood vessel growth specifically within the neocortices of *Rdh10* mutants. This phenotype was attributed to a disruption in vascular WNT signaling. In contrast, the vasculature in the nonneocortical regions (striatum and thalamus) of *Rdh10* mutants is relatively normal (Bonney et al. 2016); thus, we investigated the role of RA in BBB protein expression in these regions of wild-type and *Rdh10* mutant brains. We examined vascular Claudin-3, Claudin-5, VE-cadherin, and ZO-1 expression via immunohistochemistry (IHC). Punctate Claudin-3 expression, typical of this early developmental stage, was observed in Ib4^+^ vessels within the striatum of E13.5–E14.5 control and *Rdh10* mutants (Fig. 1*A*, *B*; arrows). At the same embryonic stages, Claudin-5 (Fig. 1*C*, *D*; arrows), VE-cadherin (Fig. 1*E*, *F*; arrows), and ZO-1 (Fig. 1*G*, *H*; arrows) were expressed and properly localized to the junctions in both control and *Rdh10* mutant Ib4^+^ vasculature. We then isolated RNA from E14.5 nonneocortical brain regions (striatum, thalamus, midbrain, and hindbrain) of control and *Rdh10* mutants and did qPCR analysis for mRNA expression of *Cldn3*, *Cldn5*, *Cdh5* (VE-cadherin), and *Tjp1* (ZO-1). *Cldn3* was significantly up-regulated (*p* = 0.0221) in *Rdh10* mutants (Fig. 1*I*). Claudin-3 is a direct transcription target of WNT signaling in brain endothelial cells. Although vascular WNT signaling is attenuated in the neocortical regions of *Rdh10* mutants, it was found that WNT signaling is significantly increased in the vasculature of nonneocortical brain regions of *Rdh10* mutants (Bonney et al. 2016). Thus the increase in Claudin-3 expression in the nonneocortical regions of *Rdh10* mutants could be due to elevated vascular WNT signaling. *Cdh5* (*p* = 0.1559) and *Tjp1* (*p* = 0.7461) gene expression were not significantly altered in *Rdh10* mutants (Fig. 1*I*). On the other hand, *Cldn5* transcript expression was significantly decreased (*p* = 0.0361) in *Rdh10* samples (Fig. 1*I*). This result is unexpected, since Claudin-5 is expressed in blood vessels of mutant brain sections (Fig. 1*C*, *D*). We next investigated BBB leakage in *Rdh10* mutants by performing IHC for fibrinogen, a serum protein that is restricted from the neural tissue by the BBB. We found retention of fibrinogen in the brain vasculature of both wild-type and *Rdh10* mutants (Fig. 1*J*, *K*; arrows), indicating that BBB function is not disrupted when RA levels are reduced. These results show that BBB function and junctional protein expression are intact in *Rdh10* mutants, indicating that reductions in RA do not disrupt BBB development and therefore RA is not required for embryonic BBB development.

### BBB protein expression is altered after *in utero* atRA exposure

**Figure 2.**
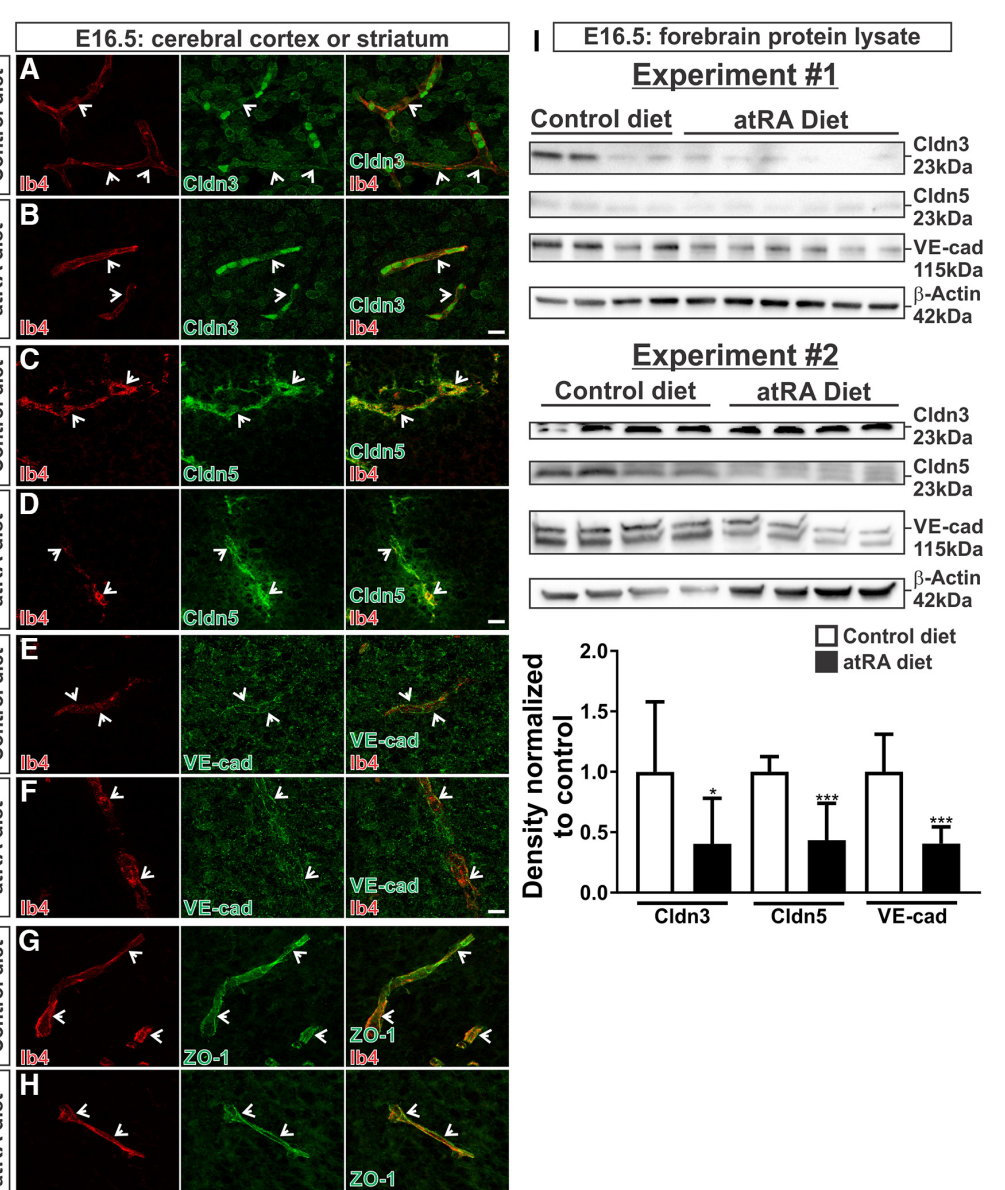
BBB protein expression is altered after *in utero* atRA exposure. Ib4^+^ blood vessels at the level of the cortex or striatum in E16.5 control and atRA-exposed brains immunolabeled with antibodies to Claudin-3 (***A*** and ***B***; green; striatum), Claudin-5 (***C*** and ***D***; green; cortex), VE-cadherin (***E*** and ***F***; green; striatum), or ZO-1 (***G*** and ***H***; green; striatum) and colabeled with Ib4 (red). Arrows indicate positive staining within vasculature for each protein. Junction formation is clear in VE-cadherin and ZO-1 staining. Scale bars are 10 µm. ***I***, Immunoblots for Claudin-3, Claudin-5, VE-cadherin, and β-actin were performed on E16.5 forebrain lysates from two different experiments of control or atRA-exposed embryos. Graph depicting densitometry (protein of interest intensity/β-actin intensity) and reduced BBB protein expression in atRA-exposed samples (*n* = 10) when normalized to control diet samples (*n* = 8). Full immunoblots are shown in Fig. 2-1. Unpaired two-tailed *t* tests were performed; *, *p* < 0.05, ***, *p* < 0.001.

To test whether RA is capable of inducing BBB properties during development, we used a prenatal RA exposure paradigm in which we fed pregnant female mice an atRA-enriched diet from E10.5 to E16.5. Based on published reports using a similar diet and quantifying atRA and 9cRA levels in the embryo (Mic et al. 2003), our atRA-enriched diet (0.175 mg/g atRA) is not predicted to generate detectable levels of 9cRA in the embryo. Thus RAR signaling, not RXR, is activated in these *in vivo* experiments. We first analyzed BBB protein expression in the CNS blood vessels of the control or atRA-exposed embryos by IHC. At E16.5, Claudin-3, Claudin-5, VE-cadherin, and ZO-1 were observed in blood vessels within the striatum (Claudin-3, VE-cadherin, ZO-1) and cerebral cortex (Claudin-5) of embryos from dams on control diets (Fig. 2*A*, *C*, *E*, *G*; arrows). In these corresponding brain regions, exposure to the atRA-enriched diet resulted in relatively normal BBB protein expression and junctional organization within the vasculature (Fig. 2*B*, *D*, *F*, *H*; arrows). However, immunoblot analysis on forebrain lysate (which includes the cortex, striatum, and thalamus) from control and atRA-exposed embryos showed a significant reduction in Claudin-3 (*p* = 0.0177), Claudin-5 (*p* = 0.0002), and VE-cadherin (*p* < 0.0001) expression after atRA exposure (Fig. 2*I*). We did note that experiment 2 displayed differential expression of β-actin after atRA exposure; however, we can find no literature that atRA directly modulates β-actin levels, and this was not observed with atRA treatment in experiment 1.

10.1523/ENEURO.0378-16.2017.f2-1 Figure 2-1 Extended data supporting Fig. 2. ***A–D***, Full immunoblots from experiment 1 of Claudin-3 (***A***; 23 kDa), Claudin-5 (***B***; 22 kDa), VE-cadherin (***C***; 115 kDa), and β-actin (***D***; 42 kDa; arrows) expression in 15 μg of forebrain lysate from embryos exposed to control or atRA diet. ***E–H***, Full immunoblots from experiment 2 of Claudin-3 (***E***; 23 kDa), Claudin-5 (***F***; 22 kDa), VE-cadherin (***G***; 115 kDa), and β-actin (***H***; 42 kDa; arrows) expression in 50 μg of forebrain lysate from embryos exposed to control or atRA diet. Protein ladders are labeled on the lefthand side of each immunoblot.. Download Figure 2-1, TIF file.

**Figure 3.**
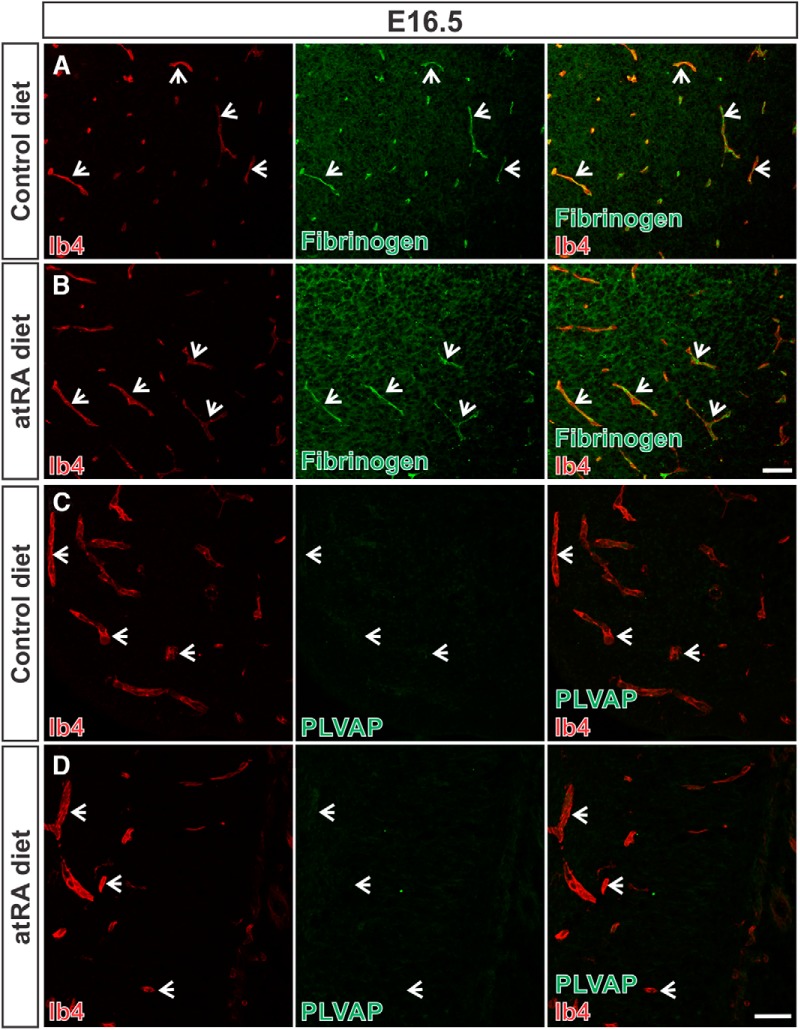
*In utero* atRA exposure does not overtly affect BBB integrity. ***A***, ***B***, Arrows show that fibrinogen (green) is contained with the lumen of thalamic vessels labeled with Ib4 (red) and not observed in the parenchyma of control and atRA-exposed E16.5 fetal brains. ***C***, ***D***, PLVAP (green; cortex) expression is absent in the Ib4-labeled vasculature (arrows) in the control and atRA-exposed E16.5 fetal brains. Scale bars are 50 µm.

We next looked for evidence of BBB disruption in the atRA-treated embryos to determine whether the reduction in Claudin-3, Claudin-5, and VE-cadherin expression affected BBB integrity. AtRA-exposed embryos showed retention of fibrinogen in the Ib4^+^ vasculature (arrows) and no leakage in the neural parenchyma (Fig. 3*A*, *B*). Additionally, we did not observe expression of another indicator of BBB breakdown, PLVAP, in the vasculature in the atRA-exposed embryos (Fig. 3*C*, *D*; arrows). Given these observations, we conclude that atRA exposure does not promote BBB features and instead may reduce BBB protein expression; however, not to the extent that BBB integrity is reduced in the prenatal brain.

### Differential effects of RA concentrations on BBB gene expression

**Figure 4.**
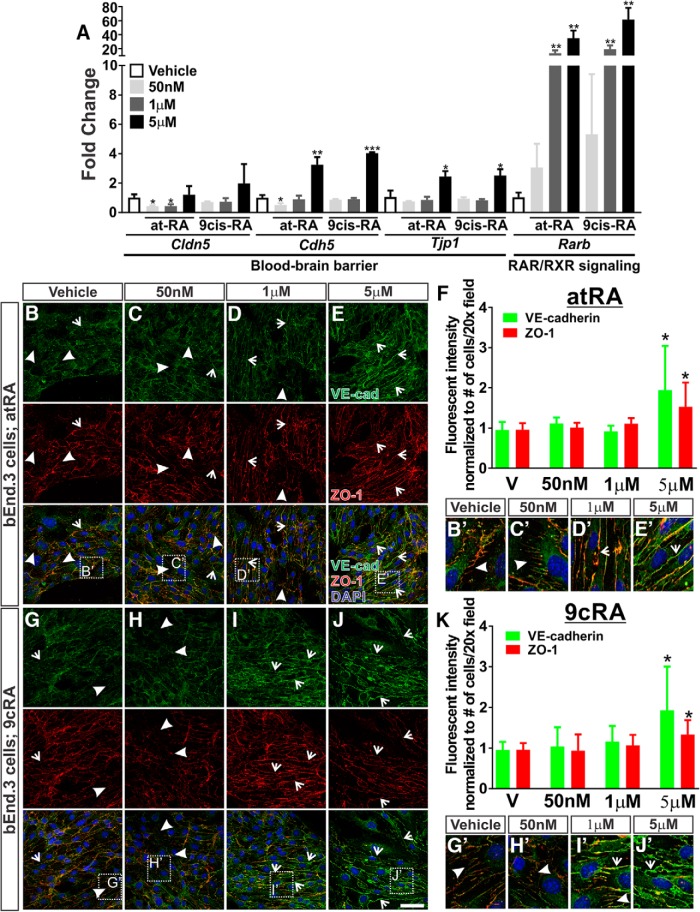
Differential effects of RA concentrations on BBB gene expression. ***A***, Representative graph depicting transcript expression of *Cldn5*, *Cdh5*, *Tjp1*, and *Rarb* normalized to *Actb* after 24 h of treatment with DMSO, 50 nm, 1 μm, or 5 μm of atRA or 9cRA show differential effects of RA concentrations in bEnd.3 cells (murine brain endothelioma cell line). Trend was consistent between all experiments (*n* = 3). All experiments (1–3) are shown in Fig. 4-1. ***B–K***, Immunocytochemistry of bEnd.3 cultures after treatments with atRA (***B–F***; DMSO, 50 nm, 1 μm, and 5 μm) and 9cRA (***G–K***; DMSO, 50 nm, 1 μm and 5 μm) for VE-cadherin (green), ZO-1 (red), and DAPI (blue) after 48 h. ***F***, ***K***, Fluorescence intensity of VE-cadherin (green) and ZO-1 (red) after treatments with atRA (***F***; DMSO, 50 nm, 1 μm, and 5 μm) or 9cRA (***K***; DMSO, 50 nm, 1 μm, and 5 μm). ***B***′***–E***′, ***G***′***–J***′, Enlarged images highlight junctional organization after treatments with atRA (***B***′: DMSO; ***C***′: 50 nm; ***D***′: 1 μm; ***E***′: 5 μm) and 9cRA (***G***′: DMSO; ***H***′: 50 nm; ***I***′: 1 μm; ***J***′: 5 μm). Arrowheads show occurrence of disjointed and disorganized junctions in physiologic concentrations of atRA and 9cRA. Arrows indicate improved junction formation in pharmacological concentrations of atRA and 9cRA. Unpaired two-tailed *t* tests were performed, *, *p* < 0.05, **, *p* < 0.01, ***, *p* < 0.001. Scale bars are 50 µm.

Recent studies showed that pharmacological concentrations of atRA (5 and 10 μm) are capable of inducing the expression of TJ and AJ genes (Mizee et al. 2013; Lippmann et al. 2014; Katt et al. 2016). Because the physiologic role of RA in BBB protein expression is unclear, we first assessed TJ and AJ gene expression after treatment with 50 nm atRA, which is within the range of atRA detected in tissues (∼2–600 nm; Napoli et al. 1991), in a murine brain endothelioma cell line (bEnd.3). bEnd.3 cells are a transformed brain endothelial cells from 6-week-old *Balb/c* mice. When used at lower passage numbers (<30), this cell line displays barrier properties, such as permeability and transendothelial electrical resistance, comparable to those of primary brain ECs. They also highly express BBB proteins such as Claudin5 and ZO-1. Therefore, these cells are a suitable alternative to primary brain ECs when studying BBB function and drug delivery owing to their rapid growth when used at appropriate passages (Brown et al. 2007; Watanabe et al. 2013). After exposure of 50 nm atRA in the bEnd.3 cells, we found that the expression of *Cldn5* (*p* = 0.0106) and *Cdh5* (*p* = 0.0159) was significantly reduced after 24 h; however, *Tjp1* (*p* = 0.2996) expression was not altered (Fig. 4*A*). This concentration of atRA was shown to inhibit WNT transcriptional activity (Bonney et al. 2016). Therefore, this effect may occur through RA-mediated suppression of endothelial WNT signaling. We next tested a high pharmacological concentration of atRA (5 µm) that has been previously shown to increase BBB gene and protein expression (Mizee et al. 2013; Lippmann et al. 2014; Katt et al. 2016). We observed that 5 μm atRA up-regulated *Cdh5* (*p* = 0.0022) and *Tjp1* (*p* = 0.0138; Fig. 4*A*). *Cldn5* expression was not significantly altered (*p* = 0.6336) after 5 μm atRA exposure (Fig. 4*A*). An intermediate concentration of atRA (1 μm) did not alter *Cdh5* (*p* = 0.5286) or *Tjp1* (*p* = 0.5246) expression; however, *Cldn5* was significantly reduced (*p* = 0.0154; Fig. 4*A*). The increase in *Tjp1* and *Cdh5* transcript expression after the pharmacological RA treatments (5 μm) in our studies using the bEnd.3 cell line follows a trend similar to that of studies using human brain ECs (Mizee et al. 2013) and iPSC-derived brain ECs (Lippmann et al. 2014; Katt et al. 2016).

10.1523/ENEURO.0378-16.2017.f4-1 Figure 4-1 Extended data supporting Figs. 4 and 5. ***A***, ***B***, Fold change expression of *Cldn5*, *Cdh5*, *Tjp1*, *Rarb*, *Gpihbp1*, *Fabp4*, *Apoe*, and *Abcg1* normalized to *Actb* in each experiment (1–3) after 24 h of DMSO, 50 nm, 1 μm, or 5 μm of atRA or 9cRA in bEnd.3 cells. ***C***, Fold change expression of *Cldn5*, *Cdh5*, *Tjp1*, *Rarb*, *Apoe*, and *Abcg1* normalized to *Actb* in each experiment after 24 h of DMSO, 5 μm atRA, 5 μm atRA + 100 nm GSK-2033 (LXR antagonist), or 100 nm GSK-2033 in b.End3 cells. ***D***, Fold change expression of *Cldn5*, *Cdh5*, *Tjp1*, *Apoe*, and *Abcg1* normalized to *Actb* in each experiment following 24 h of treatment with DMSO or 1 μm T0901317 (LXR agonist).. Download Figure 4-1, TIF file.

High concentrations of atRA have been shown to isomerize to 9cRA (Urbach and Rando 1994); therefore, the high atRA concentrations may act via 9cRA. We tested whether 9cRA is able to regulate BBB gene expression and, interestingly, 50 nm 9cRA had little effect on *Cldn5* (*p* = 0.2003), *Cdh5* (*p* = 0.3880), and *Tjp1* (*p* = 0.4259). However the pharmacological concentrations of 9cRA, similar to the effect of atRA, induced *Cdh5* (*p* = 0.0002) and *Tjp1* (*p* = 0.0349) but not *Cldn5* (*p* = 0.2750; Fig. 4*A*). Although the BBB genes showed differential responses to the RA concentrations, *Rarb*, a RAR/RXR target gene, was elevated at all concentrations with both atRA and 9cRA, with the exception of 50 nm atRA and 9cRA. However, all experiments performed showed consistent elevation of *Rarb* at all concentrations (atRA: 50 nm, *p* = 0.1003; 1 μm, *p* = 0.0078; 5 μm, *p* = 0.0051; 9cRA: 50 nm, *p* = 0.1451; 1 μm, *p* = 0.0044; 5 μm, *p* = 0.0065; Fig. 4*A*) This indicates that RAR and RXR transcriptional activity was activated at all concentrations.

We next looked at protein expression and junctional organization of ZO-1 and VE-cadherin by immunocytochemistry in the bEnd.3 cells after exposure to the physiologic (50 nm) and pharmacological (1 and 5 µm) concentrations of atRA and 9cRA for 48 h. Consistent with our transcript analysis, fluorescent intensity of VE-cadherin increased significantly after 5 μm atRA (*p* = 0.0273) and 9cRA (*p* = 0.0256; Fig. 4*E*, *F*, *J*, *K*; green) but was unaltered at lower concentrations of atRA or 9cRA. ZO-1 intensity increased after 5 μm atRA (*p* = 0.0263) and 9cRA (*p* = 0.0285; Fig. 4*E*, *F*, *J*, *K*; red); however, other concentrations of atRA or 9cRA did not have a significant effect on ZO-1 protein expression. Enlarged images revealed disjointed and disorganized junctions (as indicated by the VE-cadherin and ZO-1 staining) in the vehicle and 50 nm RA cultures (Fig. 4*B*′, *C*′, *G*′, *H*′; arrowheads); however, junctions appear improved with the pharmacological concentrations (Fig 4*D*′, *E*′, *I*′, *J*′; arrows), further suggesting an increase in junctional protein expression. These investigations corroborate previous studies showing that pharmacological concentrations of atRA (5 µm) promote BBB properties and suggest a possible role for 9cRA-RXR signaling in regulating BBB gene expression.

### Induction of BBB genes by pharmacological RA correlates with activation of LXR/RXR signaling

**Figure 5.**
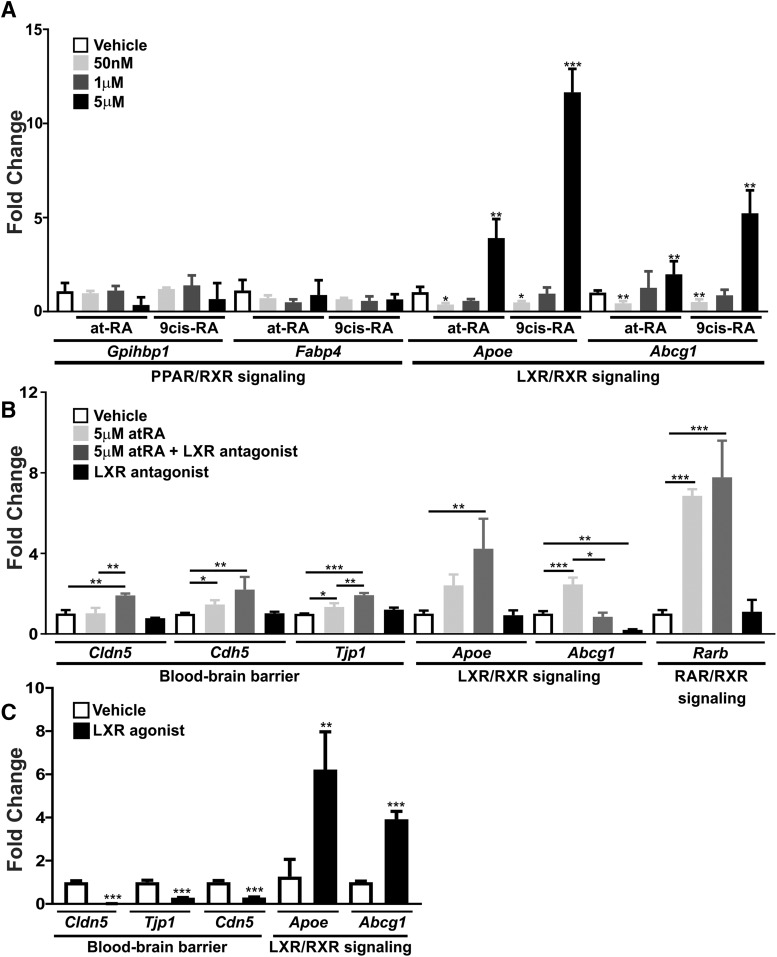
Induction of BBB genes by pharmacological RA correlates with activation of LXR/RXR signaling. ***A***, Representative graph depicting transcript expression of PPAR/RXR target genes, *Gpihbp1* and *Fabp4*, and LXR/RXR signaling genes, *Apoe* and *Abcg1*, normalized to *Actb* after 24 h of treatment with DMSO, 50 nm, 1 μm, or 5 μm of atRA or 9cRA in b.End3 cells. Graph depicts induction of LXR/RXR genes following pharmacological concentrations of RA. With the exception of *Gpihbp1* and *Fabp4*, *Apoe* and *Abcg1* expression was consistent between all experiments (*n* = 3). All experiments (1–3) are shown in Fig. 4-1. ***B***, Representative graph depicting transcript expression of *Cldn5*, *Cdh5*, *Tjp1*, *Apoe*, *Abcg1*, and *Rarb* normalized to *Actb* after 24 h of treatment with DMSO, 5 μm atRA, 5 μm atRA + 100 nm GSK-2033 (LXR antagonist), or 100 nm GSK-2033 in b.End3 cells. Inhibition of LXR signaling enhanced the induction of BBB gene expression by the pharmacological RA concentrations. Trend was consistent between all experiments (*n* = 3). All experiments (1–3) are shown in Fig. 4-1. ANOVA analysis followed by Tukey’s test was used to compare the mean values of multiple treatment conditions to one another. ***C***, Representative graph depicting transcript expression of *Cldn5*, *Cdh5*, *Tjp1*, *Apoe*, and *Abcg1* normalized to *Actb* after 24 h of treatment with DMSO or 1 μm T0901317 (LXR agonist) reveals LXR-mediated inhibition of BBB gene expression in b.End3 cells. Trend was consistent between all experiments (*n* = 3). All experiments (1–3) are shown in Fig. 4-1. Unpaired two-tailed *t* tests were performed, *, *p* < 0.05, **, *p* < 0.01, ***, *p* < 0.001.

Microarray analysis on ECs isolated from various mouse organs have shown that RXR signaling is enriched in CNS ECs (Daneman et al. 2010). Because our investigations suggested a potential role for 9cRA-RXR signaling in regulating BBB gene expression, we next investigated induction of different pathways that involve RXRs, peroxisome proliferator-activated receptor (PPAR), and liver X receptor (LXR) signaling, by pharmacological concentrations of atRA and 9cRA. Both PPARs and LXRs can heterodimerize with RXRs and regulate gene expression (Szanto et al. 2004). Treatment with the pharmacological concentrations of atRA or 9cRA did not induce two PPAR/RXR target genes, *Gpihbp1* and *Fabp4*, suggesting that PPAR/RXR signaling is not involved in the induction of the BBB genes (Fig. 5*A*). However, two LXR/RXR targets, *Abcg1* and *Apoe*, showed expression patterns similar to those of the BBB transcripts after RA treatment, where they were down-regulated with the physiologic RA concentrations (50 nm; atRA: *Apoe*, *p* = 0.0176; *Abcg1*, *p* = 0.0039; 9cRA: *Apoe*, *p* = 0.0312; *Abcg1*, *p* = 0.0097) and up-regulated with the pharmacological concentrations of atRA and 9cRA (5 μm; atRA: *Apoe*, *p* = 0.0091; *Abcg1*, *p* = 0.0011; 9cRA: *Apoe*, *p* = 0.0006; *Abcg1*, *p* = 0.0074; Fig. 5*A*). This suggests that pharmacological concentrations of RA can activate the LXR/RXR pathway in culture. Thus we hypothesized that pharmacological concentrations of RA induce the expression of BBB genes through LXR/RXR signaling. To test this, we treated bEnd.3 cultures with 5 μm atRA with and without the LXR antagonist, GSK-2033, and assessed the expression of the BBB transcripts. Unexpectedly, the addition of GSK-2033 to the 5 μm atRA–treated cultures significantly enhanced the RA-mediated up-regulation of *Tjp1* (*p* = 0.0053) compared with the 5 μm atRA samples (Fig. 5*B*). The expression of *Cldn5*, which was not induced by 5 μm atRA alone (Figs. 4*A* and 5*B* ), was significantly up-regulated with 5 μm atRA plus GSK-2033 (*p* = 0.0037) compared with vehicle-treated samples or 5 μm atRA alone (*p* = 0.0040; Fig. 5*B*). *Cdh5* was significantly elevated in cultures treated with 5 μm atRA plus GSK-2033 compared with vehicle-treated cultures (*p* = 0.0074); however, this was not significant compared with 5 μm atRA alone (*p* = 0.0740; Fig. 5*B*). *Abcg1* expression was unchanged in cultures exposed to GSK-2033 and 5 μm atRA compared with control samples (*p* = 0.8535); however, *Apoe* was significantly up-regulated in bEnd.3 cultures treated with GSK-2033 and 5 μm atRA (*p* = 0.0041). In comparison to vehicle-treated cultures, GSK-2033 did not have a significant effect on *Apoe* expression (*p* = 0.9987); however, *Abcg1* was down-regulated (*p* = 0.0098), indicating that GSK-2033 can inhibit LXR signaling (Fig. 5*B*). *Rarb* was up-regulated in cultures treated with 5 μm atRA (*p* = 0.0002) and 5 μm atRA plus GSK-2033 (*p* = 0.0001) but not with GSK-2033 alone (*p* = 0.9989) compared with vehicle-treated cultures (Fig. 5*B*). Together, these data suggest that inhibition of LXR activity with GSK-2033 enhances the RA-mediated up-regulation of the BBB genes. GSK-2033 was recently shown to augment RXR transcriptional activity (Griffett and Burris 2016); thus, it is possible that the increase in BBB gene expression is due to enhanced RXR transcriptional activation by GSK-2033. However, our data indicate that GSK-2033 alone is not sufficient to induce the expression of BBB genes (Fig. 5*B*; *Cldn5*, *p* = 0.4356; *Cdh5*, *p* = 0.9993; *Tjp1*, *p* = 0.2588) potentially because of the indirect mechanism of RXR activation. Additionally, LXRs have been known to suppress gene expression independently of RXRs, a mechanism termed LXR transrepression (Jakobsson et al. 2012). Therefore, it is also possible that GSK-2033 inhibited independent LXR activity and relieved LXR-mediated suppression of BBB gene expression. To test this, we treated bEnd.3 cells with T0901317, a potent LXR agonist. We found that *Cldn5* (*p* < 0.0001), *Cdh5* (*p* = 0.0002), and *Tjp1* (*p* = 0.0001) were significantly down-regulated in bEnd.3 cultures exposed to T0901317 (Fig. 5*C*). *Apoe* (*p* = 0.0013) and *Abcg1* (*p* = 0.0002) were significantly up-regulated, verifying activation of LXR signaling using T0901317 (Fig. 5*C*). This suggests that LXRs independently inhibit BBB gene expression. Collectively, these studies show that pharmacological concentrations of atRA can activate the LXR/RXR signaling pathway, which may be involved in the regulation of BBB gene expression observed in brain endothelial cell cultures.

## Discussion

**Figure 6.**
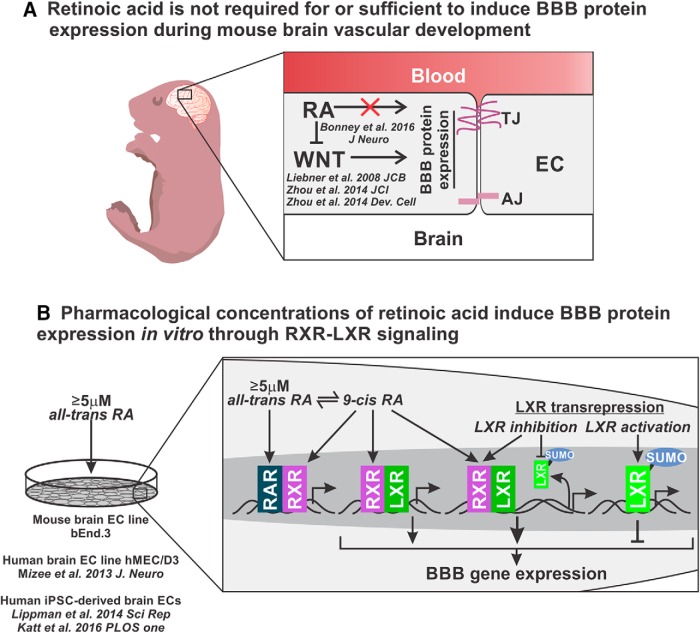
***A***, Our studies using RA-deficient embryos (*Rdh10* mutants) and atRA-exposed embryos revealed that RA is not required for or capable of enhancing BBB protein expression within the developing brain vasculature. Conversely, multiple studies show that endothelial WNT signaling is required for BBB protein expression by the developing brain vasculature (Liebner et al. 2008; Zhou and Nathans 2014; Zhou et al. 2014). A recent study also showed that RA inhibits endothelial WNT signaling, further supporting that RA does not function to promote BBB development (Bonney et al. 2016). ***B***, Recent studies have used pharmacological doses of atRA to induce BBB gene expression in human brain ECs (Mizee et al. 2013) and iPSC-derived brain ECs (Katt et al. 2016; Lippmann et al. 2014). Here we show the same effect occurs in the murine brain endothelioma cell line, b.End3 cells. Pharmacological concentrations of atRA, which activates RAR/RXR signaling, can also isomerize to 9cRA and activate the LXR/RXR signaling pathway. We also show that 9cRA is capable of inducing BBB gene expression and LXR/RXR signaling in the b.End3 cells. In our studies to identify how LXRs are involved in BBB gene regulation, we found that activation of LXR signaling using T0901317 results in the down-regulation of BBB genes. This effect may act through LXR transrepression in which LXR monomers become SUMOylated and recruit transcriptional corepressors to the promoter of BBB genes. Inhibition of LXR signaling with GSK-2033 reduces LXR SUMOylation and may relieve the LXR transrepression of BBB genes, which enhances the LXR/RXR-mediated up-regulation of BBB genes. Additionally, GSK-2033 can induce RXR transcriptional activity, thus potentially promoting the expression of BBB genes (Griffett and Burris 2016).

We have investigated the role of RA signaling in the development of the BBB and the signaling pathways activated by RA that regulate BBB gene expression in culture. We conclude that RA is not required for BBB function and the expression of BBB proteins by the developing CNS vasculature. Furthermore, excess RA does not induce BBB protein expression by the developing brain vasculature (Fig. 6*A*). Although our studies do not point to a physiologic role for RA in promoting BBB properties in the embryonic brain, we found that pharmacological concentrations of atRA (≥5 μm) up-regulate BBB gene expression and activate both RAR/RXR and LXR/RXR signaling. Although our data indicate that LXR/RXR signaling could be involved in the regulation of BBB genes, we also identified a potential role for LXR transrepression in regulating BBB properties (Fig. 6*B*).

RA has been implicated in the maturation of the BBB through regulation of BBB protein expression (Mizee et al. 2013; Lippmann et al. 2014; Katt et al. 2016). A common finding between these studies was elevated VE-cadherin and the TJ protein Occludin after pharmacological treatments of atRA in cultured human ECs. Furthermore, Mizee et al. (2013) showed that VE-cadherin transcript and BBB integrity were decreased in the fetal brain of embryos after maternal exposure to a pan-RAR inhibitor (BMS-493), which inhibits RAR transcriptional activity by promoting interactions with transcriptional corepressors. This systemic treatment, however, would block RAR transcriptional activity in neural cells, pericytes, and ECs. Although *Rdh10* mutants have diminished amounts of synthesized RA and therefore reduced RAR signaling in these different cell types, we found that VE-cadherin protein and transcript expression was not significantly altered in nonneocortical brain regions of global RA-deficient *Rdh10* mutants. This indicates that RA is not required to induce VE-cadherin in brain ECs during prenatal development. Additionally, embryos exposed to a maternal atRA-enriched diet to elevate RA levels had diminished VE-cadherin protein expression. This could be a result of decreased brain endothelial WNT signaling as has been observed with prenatal atRA exposure (Bonney et al. 2016). Although VE-cadherin interacts with WNT signaling component, β-catenin, at AJs, it is not known whether the WNT pathway directly regulates VE-cadherin expression. Alternatively, reductions in VE-cadherin expression could result directly from enhanced endothelial RA signaling. In addition to VE-cadherin, we observed reduced Claudin-3 and Claudin-5 expression, known targets of endothelial WNT signaling, in forebrain lysate of atRA-exposed embryos. Again, this is potentially due to diminished brain endothelial WNT signaling after atRA exposure. Despite alterations in VE-cadherin, Claudin-3, and Claudin-5 expression, BBB integrity was not compromised, indicating that there was sufficient expression of these and other BBB proteins such as ZO-1 to support barrier integrity.

Our analysis of *Rdh10* mutants and RA-exposed embryos indicates that the role of RA in BBB maturation is more complex than outlined in recent publications. What accounts for the different outcomes with regard to RA and BBB development? One possibility is that RA is uniquely required for later, postnatal maturation of the BBB that coincides with vessel association of astrocytic endfeet. Mizee et al. (2013) reported that human astrocytes, which they showed to express the RA-biosynthesis protein Raldh, are a possible source of the RA to induce barrier proteins. Another possibility is that the pharmacological concentrations of atRA (5 and 10 μm) used in the BBB culture models are activating several pathways in the brain ECs (Mizee et al. 2013; Lippmann et al. 2014; Katt et al. 2016), an effect we have presently tested. RARs bind to atRA with very high affinity (Delescluse et al. 1991), and the physiologic concentration of atRA is thought to be ∼25 nm within the developing mouse embryo (Mic et al. 2003). Pharmacological concentrations of atRA, however, can activate RXRs due to the isomerization of atRA to 9cRA, which is the known, high-affinity ligand for RXRs (Urbach and Rando 1994). Given that RAR activation requires concentrations of atRA in the nanomolar range, it is possible that the pharmacological concentrations of atRA used in these studies are activating RXR-mediated signaling, and this contributes to increased expression of BBB proteins and acquisition of barrier properties observed in the published culture studies.

Our studies show that high, pharmacological concentrations of atRA stimulate BBB gene expression in bEnd.3 cells, an effect that has also been observed in human brain ECs (Mizee et al. 2013) and iPSC-derived brain ECs (Lippmann et al. 2014; Katt et al. 2016). We also found that BBB gene expression can be induced with pharmacological doses of 9cRA, possibly through RXR signaling; specifically, we found activation of LXR/RXR signaling with pharmacological doses of atRA and 9cRA. Our intended use of GSK-2033 was to inhibit LXR activity to test the idea that LXR/RXR signaling activation with 5 µm RA was important for BBB gene expression. Just recently, however, GSK-2033 was shown to induce RXR, but not RAR, transcriptional activity (Griffett and Burris 2016). Therefore, our data showing enhanced BBB gene expression with 5 μm atRA and GSK-2033, a potential RXR activator, further supports the idea that RXR signaling is involved in BBB gene regulation in these *in vitro* BBB models. However GSK-2033 alone did not induce BBB gene expression, and this could be due to disparate mechanisms of RXR activation with 9cRA, atRA, and GSK-2033. Although microarray studies show enrichment of RXR signaling in brain ECs (Daneman et al. 2010), studying the role of RXR signaling in endothelial cells and BBB properties could prove difficult given the many signaling pathways RXRs are involved in and the potential for compensation from the various RXR isoforms. It is important to note, however, that pharmacological levels of RA are a useful method to enhance barrier properties in an iPSC-derived BBB model (Lippmann et al. 2014; Katt et al. 2016). This type of model has the potential to be valuable for study of drug delivery and disease. However, a more thorough understanding of the effects of high concentrations of RA and the signaling pathways involved will allow for further optimization of these BBB cell culture models. The bEnd.3 cells are an attractive cell line to model the BBB *in vitro*; however, their BBB properties (permeability and TJ expression) seem to weaken with increasing passage number (Brown et al. 2007), which is a limitation that needs to be taken in consideration when using these cells for such assays.

LXRs are activated by endogenous cholesterol metabolites such as oxysterols and sterols, which are known to up-regulate the ATP-binding cassette (ABC) transporters in brain endothelial cells. These transporters regulate cholesterol transportation and the flux of waste products out of the brain (de Wit et al. 2016). *In vivo* injury studies have shown that activation of LXR signaling using T0901317 attenuated BBB leakage in mouse models of middle cerebral artery occlusion (ElAli and Hermann 2011) and experimental intracerebral hemorrhage (Wu et al. 2016). However, this could be due to a recent phenomenon identified in innate immune cells, such as macrophages, called LXR transrepression. Upon LXR activation, LXR monomers become SUMOylated and inhibit NF-κB-, AP1-, and STAT1-mediated inflammatory responses by recruiting transcriptional repressors (Jakobsson et al. 2012). Activation of LXRs in these brain injury models could suppress a widespread inflammatory response, thus limiting BBB breakdown. Although it is unclear how LXRs function specifically in endothelial cells, our experiments with the LXR agonist, T0901317, suggest a similar mechanism whereby endothelial LXR may function independently of RXR to suppress BBB gene expression. This is potentially mediated by LXR transrepression, in which activation of LXRs increases the pool of SUMOylated LXR monomers and directly suppresses BBB gene expression (Fig. 6*B*). In addition, inhibition of LXR activity with GSK-2033 may relieve LXR-mediated suppression, therefore allowing for enhanced BBB gene expression by the pharmacological RA treatments through LXR/RXR transcriptional activity (Fig. 6*B*). Activation of LXR signaling in human umbilical vein endothelial cells (HUVECs) with various oxysterols increased endothelial barrier permeability (Hennig and Boissonneault 1987). This supports our findings that activation of endothelial LXRs may reduce barrier permeability, but it is not clear whether this effect was mediated by LXR transrepression. Later work on HUVECs and human aorta endothelial cells revealed some disparity depending on the mechanism of LXR activation. Oxysterols induced a pro-inflammatory response in endothelial cells (up-regulation of I-CAM and V-CAM; Lemaire et al. 1998); however, treatment with T0901317 attenuated the expression of the immune adhesion molecules I-CAM, V-CAM, and E-selection in lipopolysaccharide-induced inflammatory states (Morello et al. 2009). Although the role of endothelial LXRs in barrier dysfunction and immune adhesion/infiltration is unclear, these are hallmark events of many neurologic diseases and therefore understanding their role is crucial.

## Synthesis

Reviewing Editor: Maiken Nedergaard, University of Rochester Medical Center Decisions are customarily a result of the Reviewing Editor and the peer reviewers coming together and discussing their recommendations until a consensus is reached. When revisions are invited, a fact-based synthesis statement explaining their decision and outlining what is needed to prepare a revision will be listed below. The following reviewer(s) agreed to reveal their identity: Birger Brodin, Nicola Marchi

Synthesis of Reviews:

The reviewers are satisfied with your response and the manuscript is ready for publication after a minor revision. It is requested that the stats section. It is unclear whether the Authors tested for normality (parametric vs. non-parametric p).

Reviewer #1

The authors have performed new experiments, clarified statistics and modified conclusions, which have improved the manuscript greatly.

In my opinion, the authors should highlight that even the bend3 cells express junctional proteins, they do not have functional tightness (significant transendothelial resistance), which may limit the usefullness of the model. Furthermore, a bit of discussion on the use of beta-actin as reference for the westerns would be adequate (does beta actin levels stay unchanged after RA exposure).

Reviewer #2

The manuscript is greatly improved and interesting. Please expand the stats section. It is unclear whether the Authors tested for normality (parametric vs. non-parametric p).
