# Supplementary figures and images for: Differential Effects of Retinoic Acid Concentrations in Regulating Blood–Brain Barrier Properties
Source: eNeuro. 2017 May 26;4(3):ENEURO.0378-16.2017. doi: 10.1523/ENEURO.0378-16.2017 (PMC5446490; doi:10.1523/ENEURO.0378-16.2017)

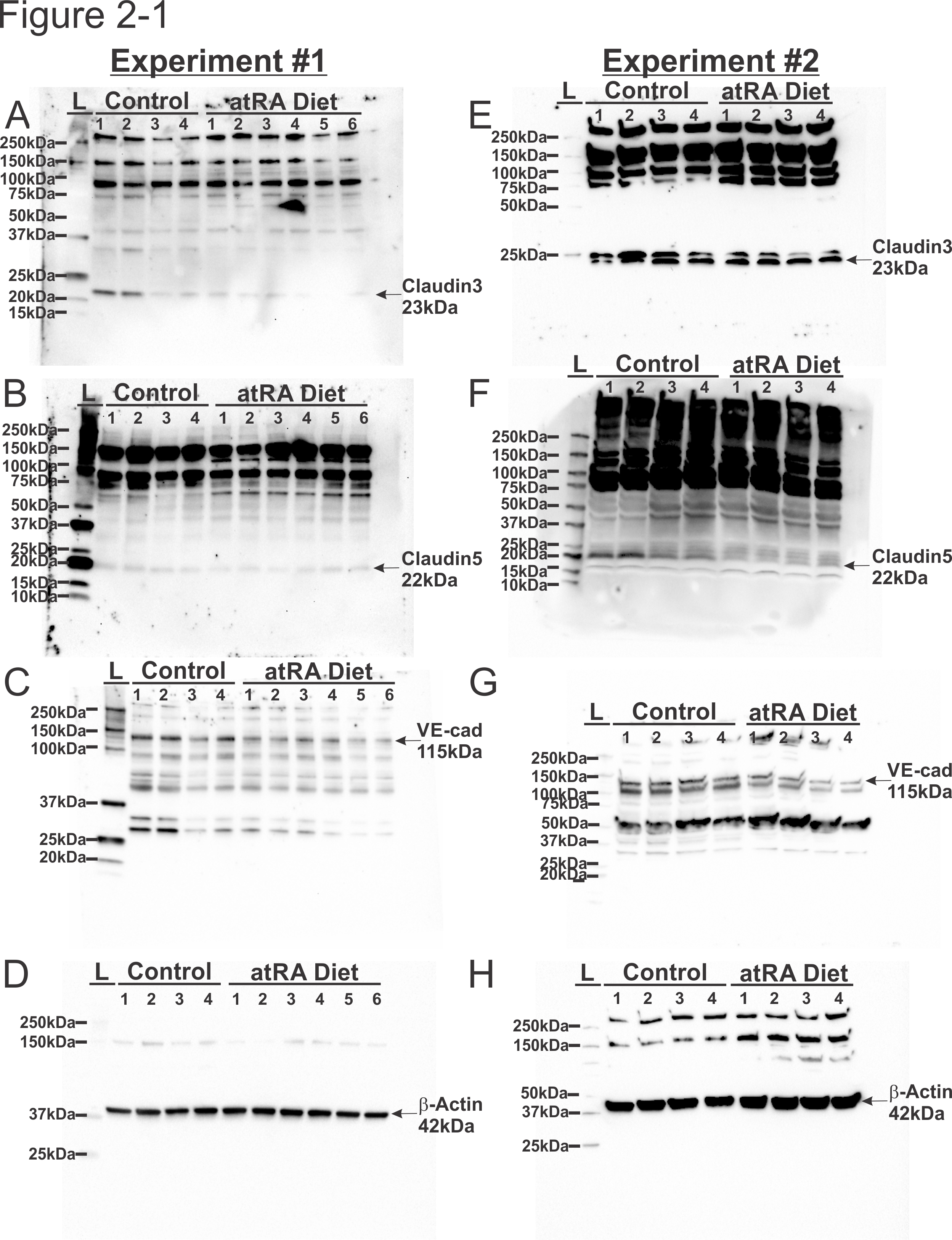

Supplement: Figure 2-1 — Extended data supporting Fig. 2. A–D, Full immunoblots from experiment 1 of Claudin-3 (A; 23 kDa), Claudin-5 (B; 22 kDa), VE-cadherin (C; 115 kDa), and β-actin (D; 42 kDa; arrows) expression in 15 μg of forebrain lysate from embryos exposed to control or atRA diet. E–H, Full immunoblots from experiment 2 of Claudin-3 (E; 23 kDa), Claudin-5 (F; 22 kDa), VE-cadherin (G; 115 kDa), and β-actin (H; 42 kDa; arrows) expression in 50 μg of forebrain lysate from embryos exposed to control or atRA diet. Protein ladders are labeled on the lefthand side of each immunoblot.. Download Figure 2-1, TIF file. [file enu003172321so1.tif]

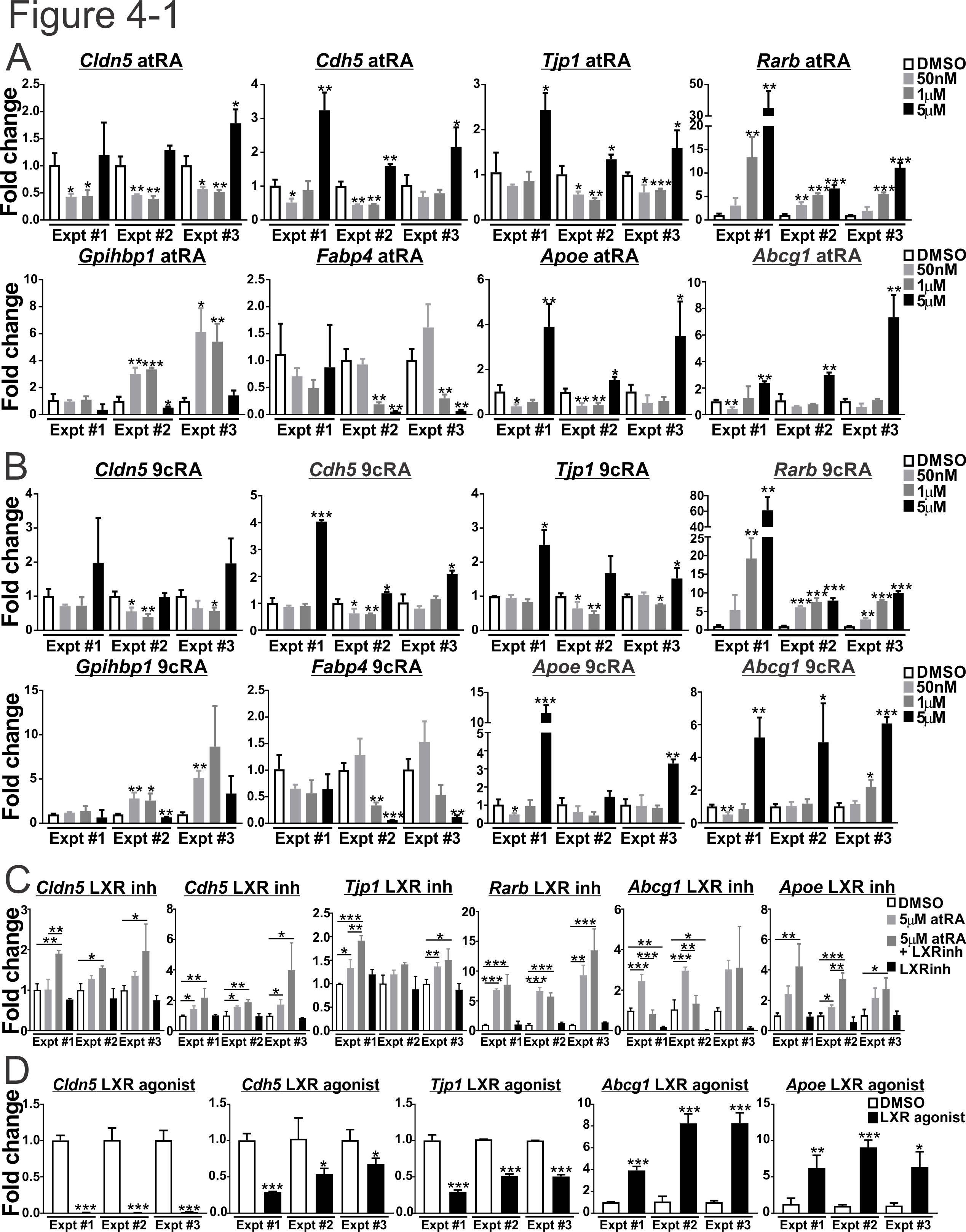

Supplement: Figure 4-1 — Extended data supporting Figs. 4 and 5. A, B, Fold change expression of Cldn5, Cdh5, Tjp1, Rarb, Gpihbp1, Fabp4, Apoe, and Abcg1 normalized to Actb in each experiment (1–3) after 24 h of DMSO, 50 nm, 1 μm, or 5 μm of atRA or 9cRA in bEnd.3 cells. C, Fold change expression of Cldn5, Cdh5, Tjp1, Rarb, Apoe, and Abcg1 normalized to Actb in each experiment after 24 h of DMSO, 5 μm atRA, 5 μm atRA + 100 nm GSK-2033 (LXR antagonist), or 100 nm GSK-2033 in b.End3 cells. D, Fold change expression of Cldn5, Cdh5, Tjp1, Apoe, and Abcg1 normalized to Actb in each experiment following 24 h of treatment with DMSO or 1 μm T0901317 (LXR agonist).. Download Figure 4-1, TIF file. [file enu003172321so2.tif]
